# Head Impact Location, Speed and Angle from Falls and Trips in the Workplace

**DOI:** 10.1007/s10439-023-03146-9

**Published:** 2023-02-06

**Authors:** Xiancheng Yu, Claire E. Baker, Mazdak Ghajari

**Affiliations:** HEAD Lab, Dyson School of Design Engineering, Imperial College London, London, UK

**Keywords:** Falls, Head impact condition, Work-related injury, Helmet standards, Multi-body dynamics, Brain injury

## Abstract

Traumatic brain injury (TBI) is a common injury in the workplace. Trips and falls are the leading causes of TBI in the workplace. However, industrial safety helmets are not designed for protecting the head under these impact conditions. Instead, they are designed to pass the regulatory standards which test head protection against falling heavy and sharp objects. This is likely to be due to the limited understanding of head impact conditions from trips and falls in workplace. In this study, we used validated human multi-body models to predict the head impact location, speed and angle (measured from the ground) during trips, forward falls and backward falls. We studied the effects of worker size, initial posture, walking speed, width and height of the tripping barrier, bracing and falling height on the head impact conditions. Overall, we performed 1692 simulations. The head impact speed was over two folds larger in falls than trips, with backward falls producing highest impact speeds. However, the trips produced impacts with smaller impact angles to the ground. Increasing the walking speed increased the head impact speed but bracing reduced it. We found that 41% of backward falls and 19% of trips/forward falls produced head impacts located outside the region of helmet coverage. Next, we grouped all the data into three sub-groups based on the head impact angle: [0°, 30°], (30°, 60°] and (60°, 90°] and excluded groups with small number of cases. We found that most trips and forward falls lead to impact angles within the (30°, 60°] and (60°, 90°] groups while all backward falls produced impact angles within (60°, 90°] group. We therefore determined five representative head impact conditions from these groups by selecting the 75th percentile speed, mean value of angle intervals and median impact location (determined by elevation and azimuth angles) of each group. This led to two representative head impact conditions for trips: 2.7 m/s at 45° and 3.9 m/s at 75°, two for forward falls: 3.8 m/s at 45° and 5.5 m/s at 75° and one for backward falls: 9.4 m/s at 75°. These impact conditions can be used to improve industrial helmet standards.

## Introduction

Traumatic brain injury (TBI) is a common injury all over the world with significant socioeconomical costs.^37^ An estimated 57 million TBIs occur worldwide annually.^25^ In the US alone, approximately 1.7 million people are diagnosed with TBI each year and the financial loss is over $75 billion.^12,37^ Industrial workers are at significant risk of TBIs. According to a recent database analysis, workplace or work-related TBIs (wr-TBIs) account for 17.9% of all TBIs.^41^ In the United Kingdom alone, there were 348,453 work-related TBI cases admitted to hospital in 2019–2020.^19^ It is likely that the problem is far worse due to poor reporting. A survey conducted by Headway, a UK charity, found that 52% of workers who had experienced a head injury did not report it to their manager, with only 6% of those injured seeking medical attention.^19^ Wr-TBIs are caused by falls, vehicle collisions, being struck by/against an object and assaults.^9^ Falls have been identified as the leading cause of wr-TBI, accounting for 50% to 70% of all wr-TBI cases.^21,24,27^ In addition, falls particularly falls from height have been shown to produce more severe TBIs and more fatalities than any other mechanisms.^8,22^

Industrial safety helmets are worn to protect the head against wr-TBI. They are designed to pass standards. Current standards (e.g., EN379 in Europe and Z89 in the US) mandate two types of impact tests both representing a falling object. The helmet is fitted onto a headform that is fixed to the base. A flat or sharp object is dropped on the helmet and the force under the headform is measured.^5^ Hence, these tests assess helmet’s ability in protecting the head against falling objects, which are the cause of TBI in 17% of the cases only. There is a need to test helmets under conditions that represent falls.^8^ In addition to local forces and head translation, falls are likely to produce significant head rotation, which can damage axons and vessels in the brain and lead to long-term effects.^8,16,23,46^

An impediment to designing more representative test conditions is the lack of understanding of the head impact conditions in work environments. Several challenges contribute to this. A primary challenge is the wide range of possible impact scenarios and the difficulty in categorising them. The conditions prior to the impact, such as worker size, body posture, self-balancing, bracing actions, nearby obstruction etc., vary largely across accidents. Small differences in these conditions can lead to very different head impact conditions. In addition, the impact medium can be any structure and object on site. These are different to road traffic collisions (RTCs), where the initial body gestures are mostly walking, sitting or riding and the impact medium is mostly road and vehicle surface.^10,38^ The lack of CCTV video footage further increases the difficulty in understanding the scenarios of workplace accidents. Compared with RTCs, head impacts in workplace are rarely captured by CCTV and the details of the accident rely heavily on the victim and witness's memory, which can be inaccurate.

One approach to overcome some of these challenges and improve our understanding of workplace head impacts is computational modelling. Previous studies have simulated falls in work environment using multi-body dynamics.^1,28,29,32^ These studies provided new information about the head impact kinematics resulting from falls. However, they focused on reconstructing individual cases in which the conditions prior to the fall are prescribed according to case reports. These initial conditions can influence the head impact speed, angle and location, the parameters that are needed for designing representative lab tests. Hence, there is a need to study the effects of initial fall conditions on the head impact speed, angle and location and determine head impact conditions representative of the wr-TBI scenarios.

In this study, we predicted the head impact conditions from a large range of simulated trips and falls in the workplace. We used multi-body computational modelling to simulate falls. One of our aims was to determine the effects of worker size, initial posture, falling height, tripping barrier width and height and bracing on the head impact location, speed and angle. Our other aim was to determine representative test conditions which can be used to design better impact test methods for industrial safety helmet standards.

## Methods

**FIGURE 1:**
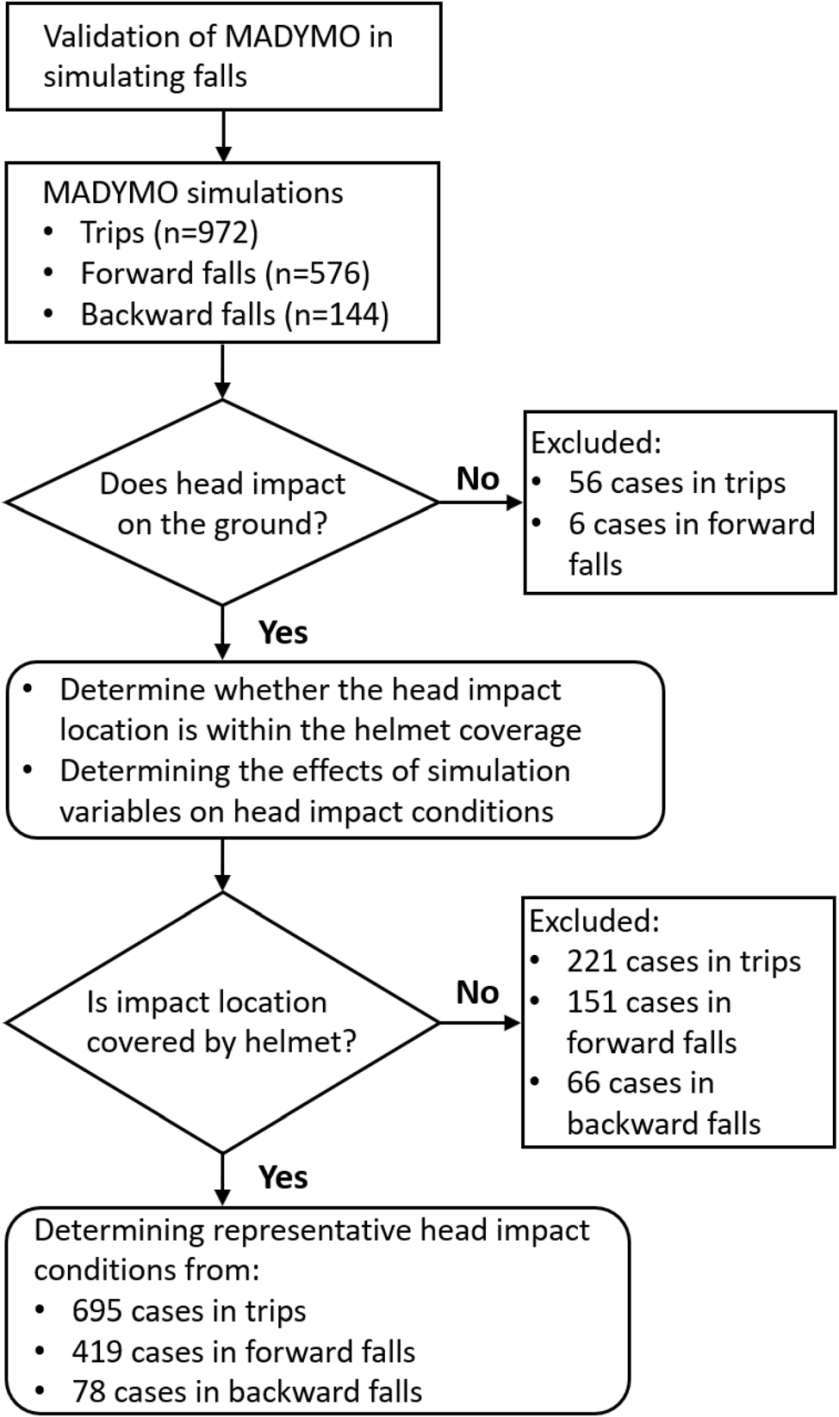
A flow chart showing the methods and inclusion criteria for the three simulated fall scenarios.

We simulated three fall scenarios: trips representing a fall onto the same level, forward falls and backward falls both representing falls onto a lower level. We used the MADYMO multi-body dynamics software to simulate the falls. The human body models in MADYMO have been extensively validated against volunteers and postmortem human subjects (PMHS) impact tests, showing reliable and good prediction of the kinematics and dynamic response of human body.^17,18,38^ The human body models have been used in several previous studies that studied human body kinematics and biomechanics in falls, including our recent work on falls of e-scooter riders.^1,13,29,33^ Figure 1 shows an overview of the simulations. We first validated the prediction of our model against a fall accident, which is a backward fall case. Then, we simulated the three fall scenarios, including forward falls, backward falls and trips. It should be noted that the MADYMO model was not validated for forward falls and trips, as we did not find suitable video footage for these accidents. We processed the results to check whether a head-ground impact was detected and exclude cases without head-ground impact. The impact location, speed and angle were determined for each case with a head-ground impact. These results were further analysed to determine the effects of the simulation variables, such as worker size and body posture prior to trips and falls, on the head impact speed and angle. Next, we further excluded cases where head impact location was outside the helmet coverage and determined representative head impact conditions for helmet impact tests from the remaining cases.

### Validation of the Multibody Dynamics Model

We validated the MADYMO model against a fall from height accident captured by a CCTV camera. A male worker lost balance when climbing a ladder on a steel platform. The worker first landed on the platform and continued moving backwards, falling from the platform. The worker experienced a free fall and landed on the ground with contact points on his back and the rear of his head. We simulated the free fall phase of the accident using the 50th percentile male ellipsoid pedestrian model of MADYMO. We estimated the falling height to be 3.5 m, using the 50-L compressed gas cylinder (the bule colour cylinder under the platform) as a landmark. We adjusted the model’s initial body gesture and position based on the CCTV footage.

We used two approaches to validate the simulation. First, we compared the time that took the worker to fall onto the ground from the platform between the MADYMO simulation and the CCTV footage. Second, we compared the body posture predicted from the simulation and the footage.

### The Simulated Falls

**FIGURE 2:**
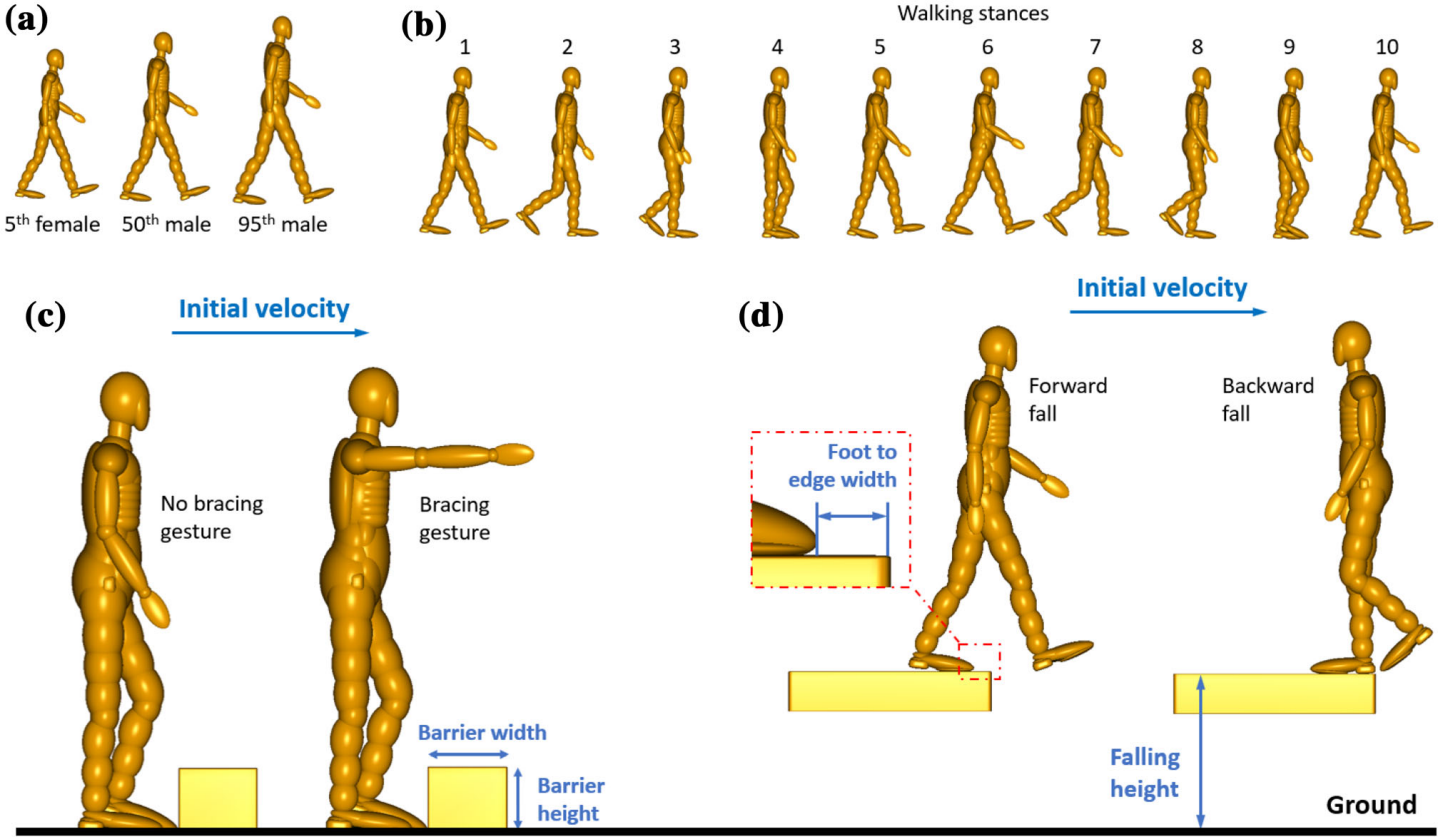
MADYMO simulation conditions: (a) human model with different size, (b) human walking stances, (c) conditions in trips simulation and (d) conditions in falls simulations.

The three simulated fall scenarios are trips, forward falls and backward falls (Figs. 2c and 2d). For each scenario, we used three human body models, 5th percentile female (5F), 50th percentile male (50 M) and 95th percentile male (95 M), representing a large population of industrial workers (Fig. 2a).

Walking stance may affect the body kinematics during falls. It can be divided into 10 stances, as shown in Fig. 2b.^14,42^ It should be noted that only some walking stances may lead to falls. For example, most trips happen when one leg moves across the other stationary leg. Therefore, we simulated stances #4, #5 and #6 when left leg crosses the right leg. For falls onto a lower level, we selected stances #1 and #3 to simulate forward fall and backward fall, respectively. Stance #3 has a smaller leg-opening than #1 as the backward walking has smaller stride length than forward walking.^26^

The mean comfortable walking speed for men and women between their 20s and 50s ranges from 1.39 to 1.46 m/s while the mean maximum walking speed for the same age groups ranges from 2 to 2.5 m/s.^4^ Therefore, we used six walking speeds for trips and forward falls simulations: 1, 1.2, 1.4, 1.6, 1.8 and 2 m/s. For backward falls, we used a stopwatch to measure the backward walking speed of several volunteers and selected six values: 0.2, 0.4, 0.6, 0.8, 1 and 1.2 m/s.

For trips, the barrier width and height may affect the body kinematics. We modelled a 1 m-long rectangular barrier and varied its width and height. The arm bracing gesture may also affect the body kinematic in trips.^31^ We simulated two arm gestures: arms to the side resulting in no bracing against the fall and arms held out to brace against the fall (Fig. 2c).

For falls to a lower level, we varied the falling height for both forward and backward falls. We varied the foot-to-edge distance as it has been shown to affect the head impact conditions.^1^ For forward falls, we varied this distance from 0 to 0.3 m due to the large separation angle of the legs in stance #1. For backward falls, we fixed the distance to 0 due to the small separation angle of the legs in stance #3. For falls to a lower level, we did not consider arm bracing gesture as arm gesture is difficult to predict during free falls.

The impact parameters are listed in Table 1. In total, 1692 simulations were conducted for the three scenarios (972 trips, 576 forward falls to lower level and 144 backward falls to lower levels). It should be noted that we did not augment the simulation conditions by mirroring the stances, i.e., doubling the number of simulations by adding the opposite stances. This is because doing this will produce head impacts with the same impact speed and angle, but mirrored head impact location about the sagittal plane. When determining the average head impact locations, this symmetry will lead to head impacts being located exactly on the sagittal plane, which is unrealistic.

**TABLE 1:** The variables for the three simulated scenarios.

Variables	Trips (n = 972)	Forward falls to lower level (n = 576)	Backward falls to lower level (n = 144)
Worker size	5th female, 50th male, 95th male	5th female, 50th male, 95th male	5th female, 50th male, 95th male
Walking stance	#4, #5, #6	#1	#3
Walking speed (m/s)	1, 1.2, 1.4, 1.6, 1.8, 2	1, 1.2, 1.4, 1.6, 1.8, 2	0.2, 0.4, 0.6, 0.8, 1, 1.2
Arm bracing	Yes, No	No	No
Tripping barrier width (m)	0.1, 0.2, 0.3	–	–
Tripping barrier height (m)	0.15, 0.25, 0.35	–	–
Falling height (m)	–	0.5, 1, 1.5, 2, 2.5, 3, 3.5, 4	0.5, 1, 1.5, 2, 2.5, 3, 3.5, 4
Foot to edge distance (m)	–	0, 0.1, 0.2, 0.3	0

### The Simulation Setup

The contact characteristics of the MADYMO simulation is based on the default force–deflection curve defined for each part of the human body model. These contact characteristics have been validated against full body pedestrian impacts. The coefficient of friction (CoF) between the human model and the floor surface and the barrier was set to 0.67, as suggested in previous studies.^30,39,40^ This value was determined by averaging experimental measurements of CoF between human body and ground surface with various conditions (e.g. dry and wet).^44^ The simulation time was set to 2.5 s, which is long enough to capture the details of the head-ground impact. All simulations were executed using the MADYMO solver version 2021.1

### Outcome Measures and Statistical Analysis

**FIGURE 3:**
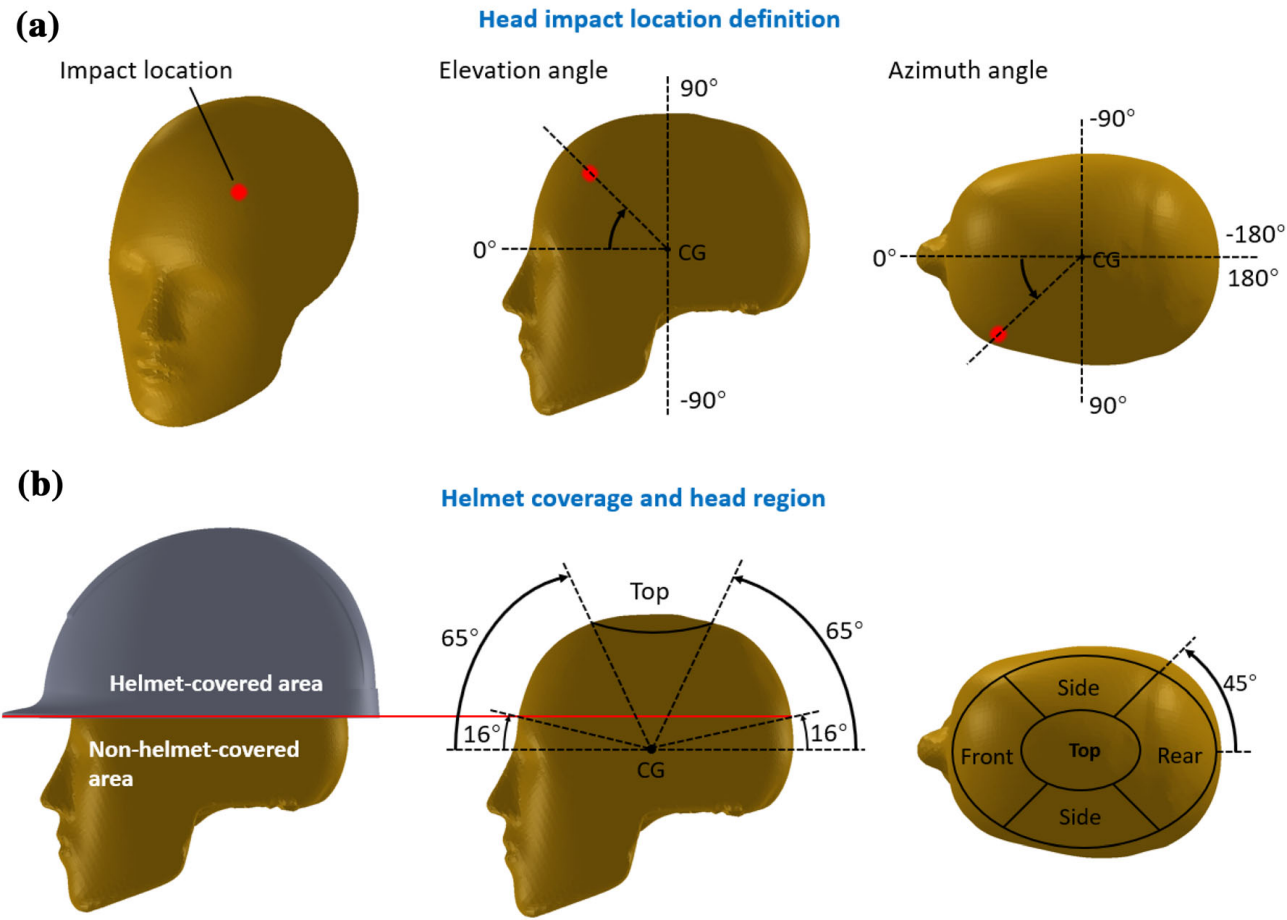
(a) Definition of head impact locations, using elevation and azimuth angle. (b) Impact location grouped as helmeted covered area (elevation angle > 16°) and uncovered area (elevation angle < 16°). The helmet-covered area was further divided into four regions: front, side, rear and top.

We processed simulation results to determine the head impact speed and angle to the ground and the impact location on the head. The head impact speed and angle results were written into two csv files by the MADYMO solver. We developed an in-house Python code to detect the time of head-ground impact, which was used to find the corresponding values in the csv files. The impact location was defined by two angles: elevation angle and azimuth angle (Fig. 3a). The head rotation about its local coordinate system was recorded during the simulation. By using the rigid body rotation theory, we were able to determine the head orientation at the time of impact. As the ground is horizontal, the elevation and azimuth angles of the impact location were calculated from the head orientation.

We determined the helmet coverage area by defining a threshold for the elevation angle. There are several types of industrial safety helmets with different configurations used for different workplace activities (e.g. an additional visor for eye protection). Currently, the most widely used helmets in industrial workplace are the standard safety helmets, which have an injection-moulded polymer shell and a harness suspension. To determine the helmet coverage, we select one of such helmets (JSP EVO3 linesman safety helmet), which is made by a leading safety helmet manufacturer (JSP) and is widely available in the UK market. We fitted the helmet onto a 50th Hybrid III (HIII) dummy headform and measured the elevation angle of the helmet edge (Fig. 3b). The helmet covered the head starting from an elevation angle of 16°. We counted the number of impacts which were in the area covered by the helmet and in the areas uncovered by the helmet. In addition, the helmet covered area was further divided into five regions, front, left side, right side, back and top, and the number of impacts in each region was counted (Fig. 3b).

For each impact scenario, one-way ANOVA was used to determine the effects of different simulation variables (e.g., worker size) on the outcome measures. The ANOVA results were reported as F-value (degrees of freedom of independent variables, residuals) and *p*-value in the appendix (Table 3). The statistical analysis was performed in the open-source software R.

### Determining the Representative Head Impact Conditions

The head impact conditions include four parameters: impact speed, impact angle, the elevation angle and the azimuth angle of the impact location. The simulations showed that larger impact speeds were associated with larger impact angles, i.e., impact velocities being more perpendicular to the ground. Therefore, we grouped the results into three groups of impact angles: [0°–30°], [30°–60°] and [60°–90°]. First, we excluded groups that had very few cases. Then, we plotted the impact speed and impact location (elevation and azimuth angles) with respect to these three groups. For each group, we determined the 75th percentile value of impact speed. Rowson *et al*. also considered a 75th percentile threshold of head kinematic metrics (in addition to 25th percentile) when assessing concussion injury risk.^35^ Unfortunately, there are no injury risk curves directly available to relate head impact speed (in falls or otherwise) to clearly classified injury outcomes, such as the Abbreviated Injury Scale (AIS). However, head impact speed in pedestrian-vehicle collisions has been shown to relate very closely to vehicle impact speed,^14^ which has been shown to be a significant predictor of head injury outcome.^2,48^ Therefore, we can deduce that higher head impact speed will produce more severe head injury irrespective of classification. Using the 75th percentile speed allowed us to cover at least 75% severity of these cases. The 50th percentile value of elevation and azimuth angles represented the most frequently impacted location. The impact angle was selected at the middle point of each group (e.g., 45° for group [30°–60°]). This approach allows for easy adaption of test rigs which are frequently used in helmet impact studies.

## Results

### The Multibody Dynamics Model Predicts the Fall Time and Head Impact Location

**FIGURE 4:**
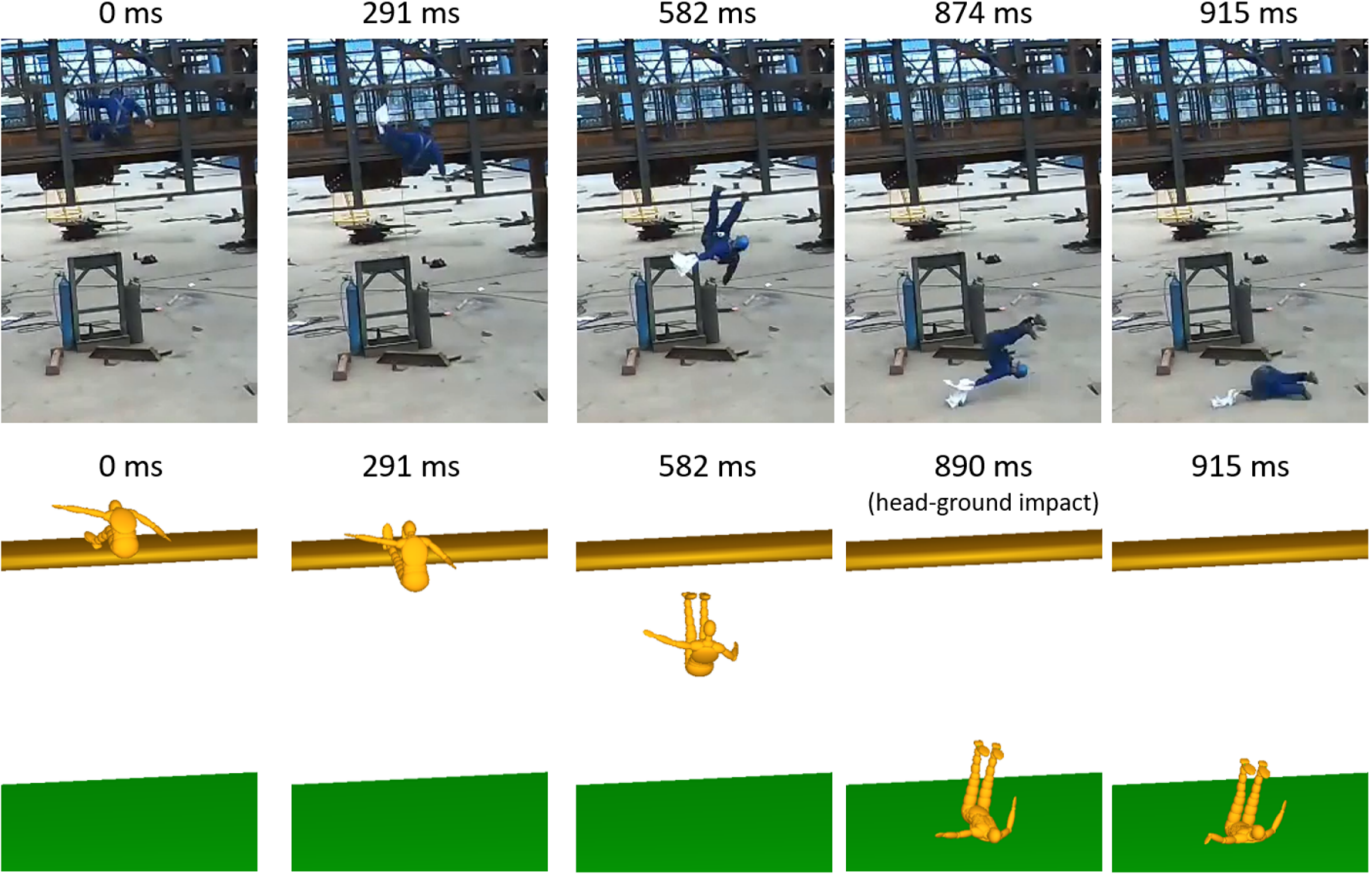
Prediction of a fall from height using multibody dynamics modelling. The predicted head-ground impact time and location agree well with the video footage.

Figure 4 shows the validation results of the MADYMO simulation against the video footage of the fall accident. The CCTV footage was recorded at 24.03 frames per second, corresponding to 41.6 ms per frame. The head-ground impact happened between 874 (frame 22) and 915 ms (frame 23) from the initial state. The simulation predicted that the head-ground impact occurred at 890 ms from initial state, which is in good agreement with the video footage.

Next, we compared the predicted body kinematics with the CCTV footage. From the visual comparison, there is a good agreement in human body posture between the CCTV footage and MADYMO prediction up to 582 ms (Fig. 4). After this time, we observed differences between the motion of the worker and the model prediction (e.g. body orientation and posture), which can be attributed to the fact that the worker bends his upper body to keep balance and avoid head in the downward orientation, but the dummy does not.^38^ In addition, the initial feet-platform interaction may also contribute to the large body rotation of the worker (about the normal to the ground), which is difficult to model in the MADYMO simulation. The final head impact location was predicted correctly, which was at the back of the head. The head-ground impact speed was predicted to be 9.5 m/s. These comparisons provide an initial validation for the predictive capabilities of the MADYMO simulations for our study.

### Head Impact Location

**FIGURE 5:**
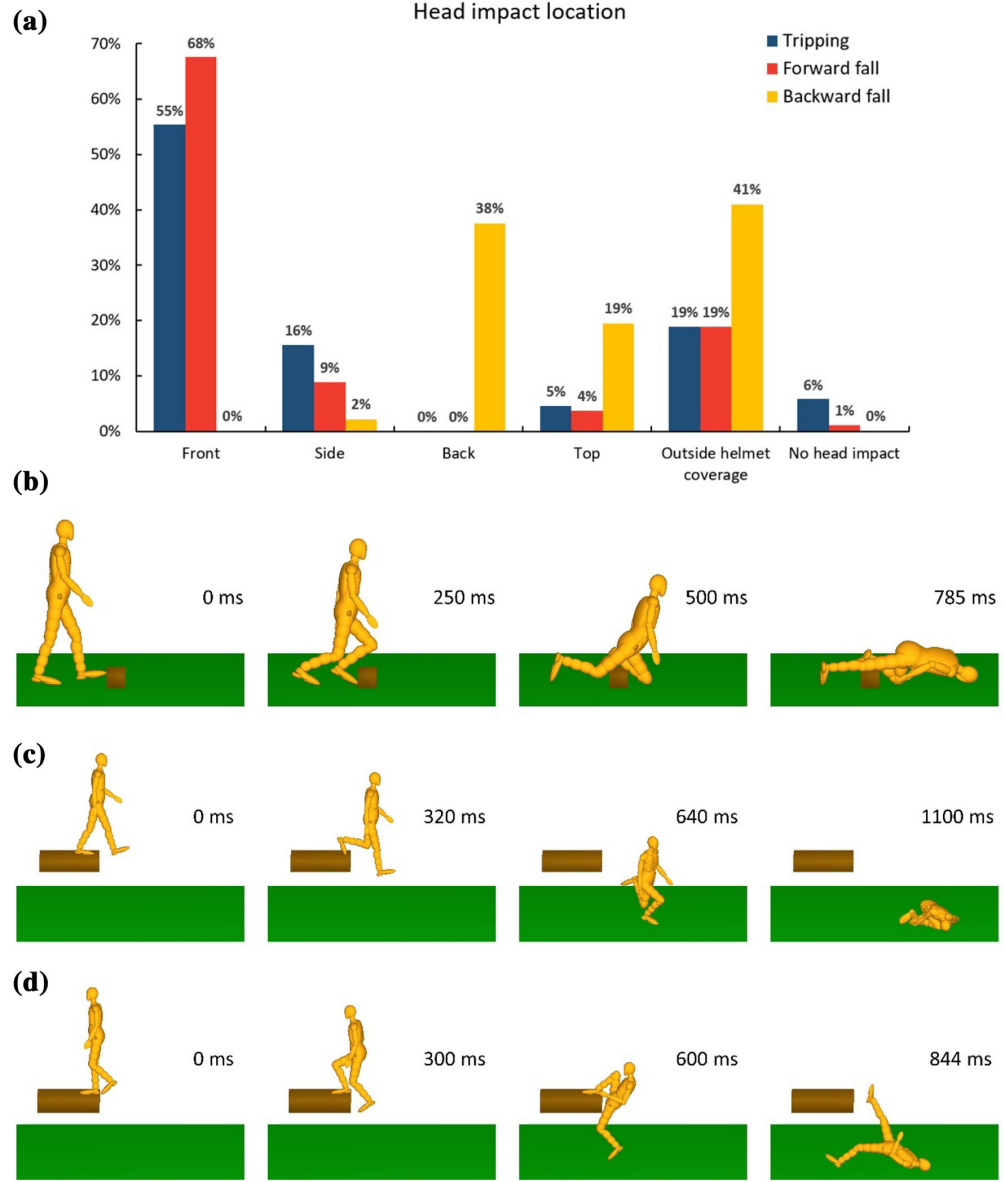
(a) Head impact location predicted by MADYMO simulations. (b) MADYMO simulation of a trip (size: 50th male, stance: #5, no arm bracing, walking speed: 1.6 m/s, barrier width: 0.2 m, barrier height: 0.15 m). (c) MADYMO simulation of a forward fall (size: 50th male, stance: #1, no arm bracing, walking speed: 1 m/s, falling height: 1 m, foot-edge distance: 0.1 m). (d) MADYMO simulation of abackward fall (size: 50th male, stance: #3, no arm bracing, walking speed: 0.4 m/s, falling height: 1 m, foot-edge distance: 0 m).

Figure 5a shows the location of head impact of the three simulated scenarios. Figures 5b–5d show examples of the predicted body motion for each scenario. In the trip scenario, most impacts occurred at the front (55%), followed by side (16%) and top (5%). No impact was observed at the back region. In addition, 6% of the trips did not lead to direct head-ground impact due to the head impacting the arm. In the forward fall scenario, 68% of the impacts were at the front, followed by side (9%) and top (4%). Only 1% of the cases did not lead to direct head-ground impact. In contrast, most backward falls resulted in impacts at the back (38%) and top (19%), with only 2% of the impacts to the side and none to the front. A large percentage of impacts were outside the area of helmet coverage. This was 41% for the backward falls and 19% for both the trips and forward falls. For the cases with head impacts outside the helmet coverage area, we plotted the elevation and azimuth angles of the impact locations with respect to the worker size in the appendix (Fig. 9).

### The Effects of Simulation Variables on the Head Impact Speed and Angle

**FIGURE 6:**
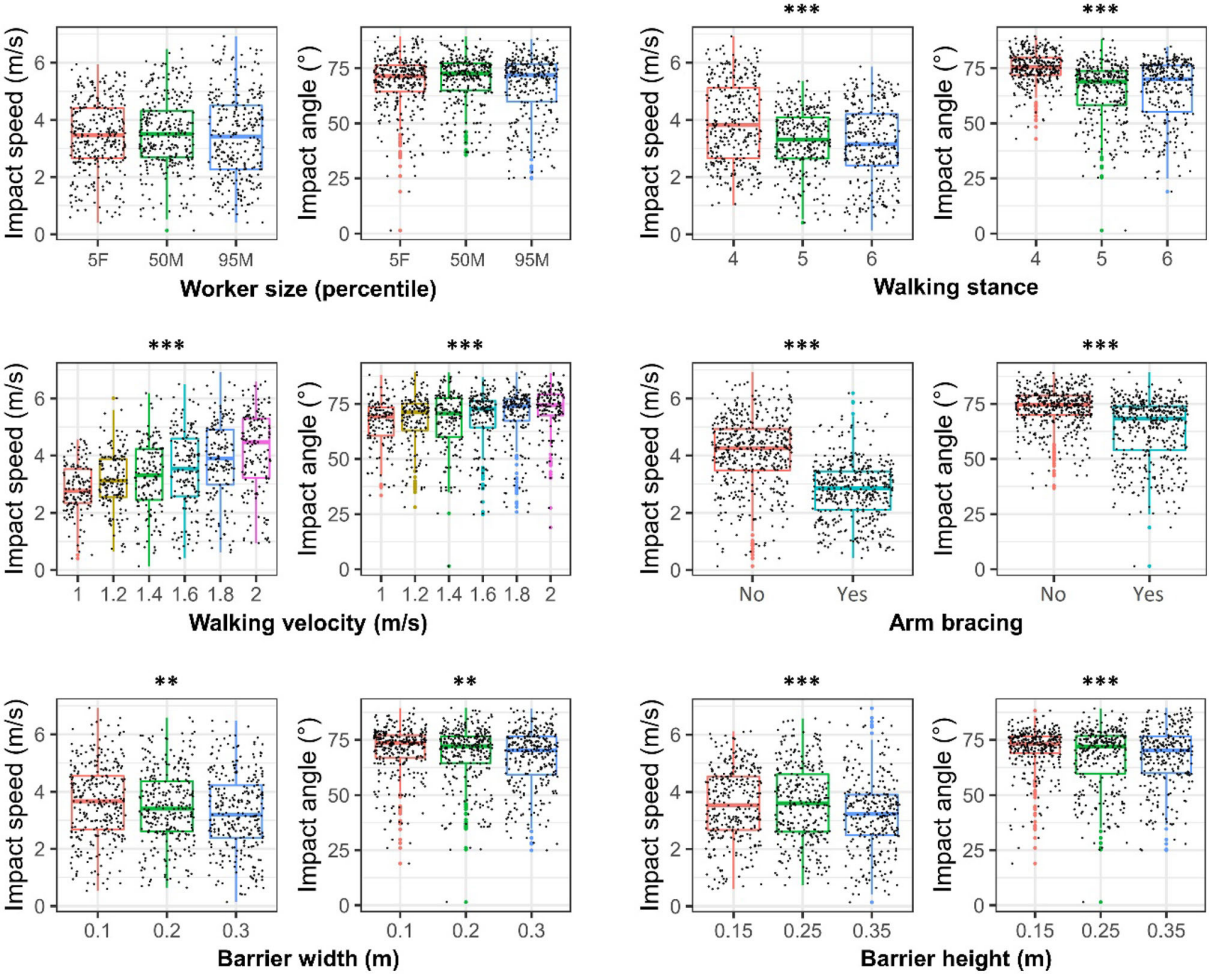
The effects of simulation variables on the head-ground impact speed and angle of trip scenarios. (The one-way ANOVA results are represented at the top of each figure: ***p < 0.001, **p < 0.01, *p < 0.05).

In trips, the predicted impact speed and angle were 3.5 ± 1.3 m/s and 68.5° ± 12.9°. The worker size did not have a significant effect on the impact speed and angle (Fig. 6). The other simulation variables had a significant effect on the impact speed and angle. Specifically, the arm bracing gesture reduced the median impact speed by 1.43 m/s and impact angle by 6.38° compared to no arm bracing. In addition, with higher walking speed, the head-ground impact speed and angle were higher.

**FIGURE 7:**
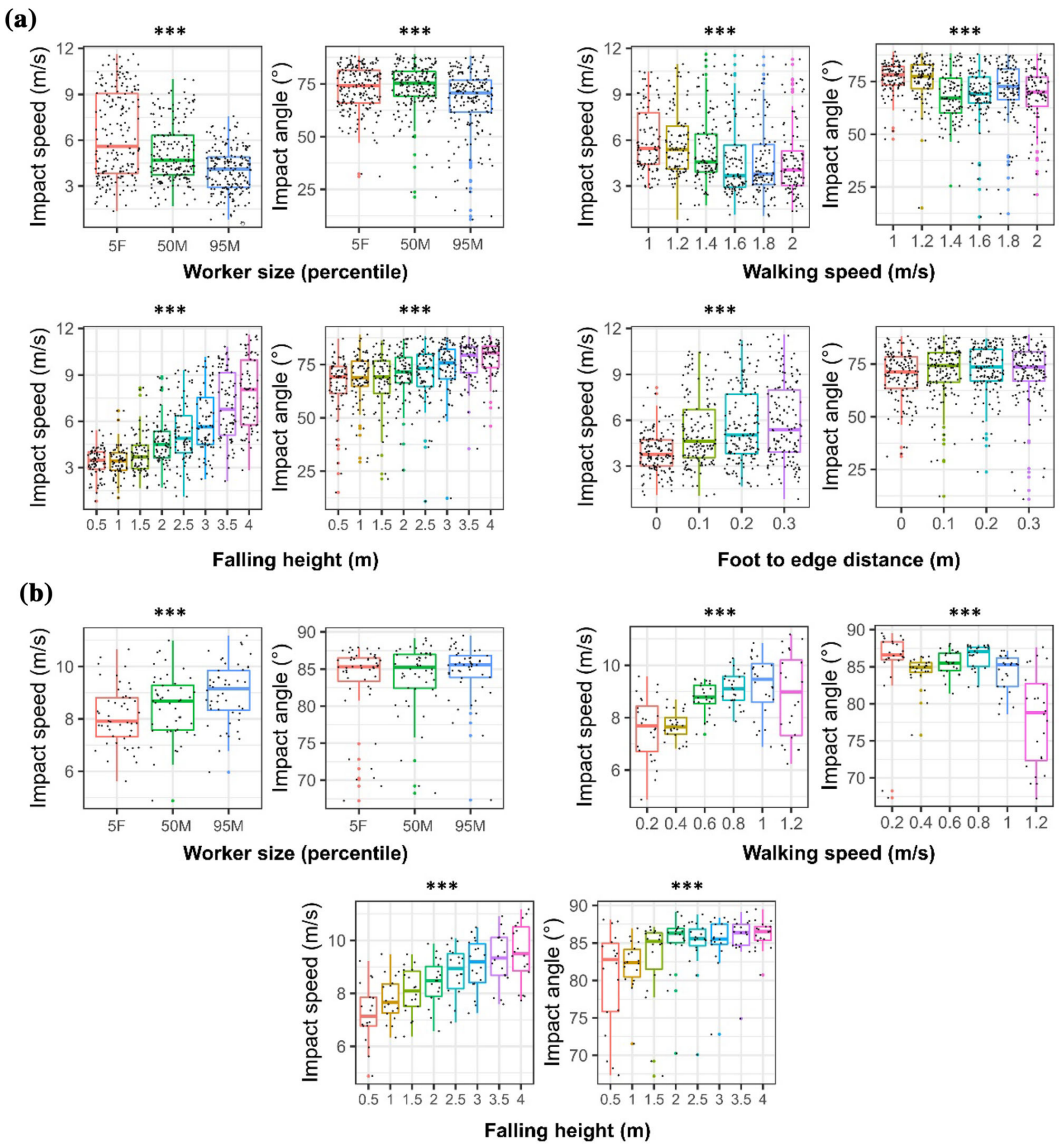
The effects of simulation variables on the head-ground impact speed and angle of (a) forward and (b) backward fall scenarios. (The one-way ANOVA results are represented at the top of each figure: ***p < 0.001, **p < 0.01, *p < 0.05).

For forward falls, the predicted impact speed and angle were 5.2 ± 2.3 m/s and 71.3° ± 12.5°. The one-way ANOVA showed that only the foot-edge distance did not have a significant effect on head impact angle (Fig. 7a). All the other simulation variables significantly affected the head-impact speed and angle. Interestingly, increasing the worker size and the walking speed resulted in a reduction in head impact speed. The falling height shows a prominent effect on impact speed. The median head impact speed produced by a 4 m-fall is 2.5 times of that produced by a fall from a 0.5 m height.

For backward falls, the predicted impact speed and angle were 8.5 ± 1.2 m/s and 84.0° ± 4.7°. All simulation variables, except from the worker size, had a significant effect on the head impact angle (Fig. 7b). Similar to the forward falls, the falling height had a prominent and consistent effect on head impact speed.

### Representative Head Impact Conditions for Future Test Standards

**FIGURE 8:**
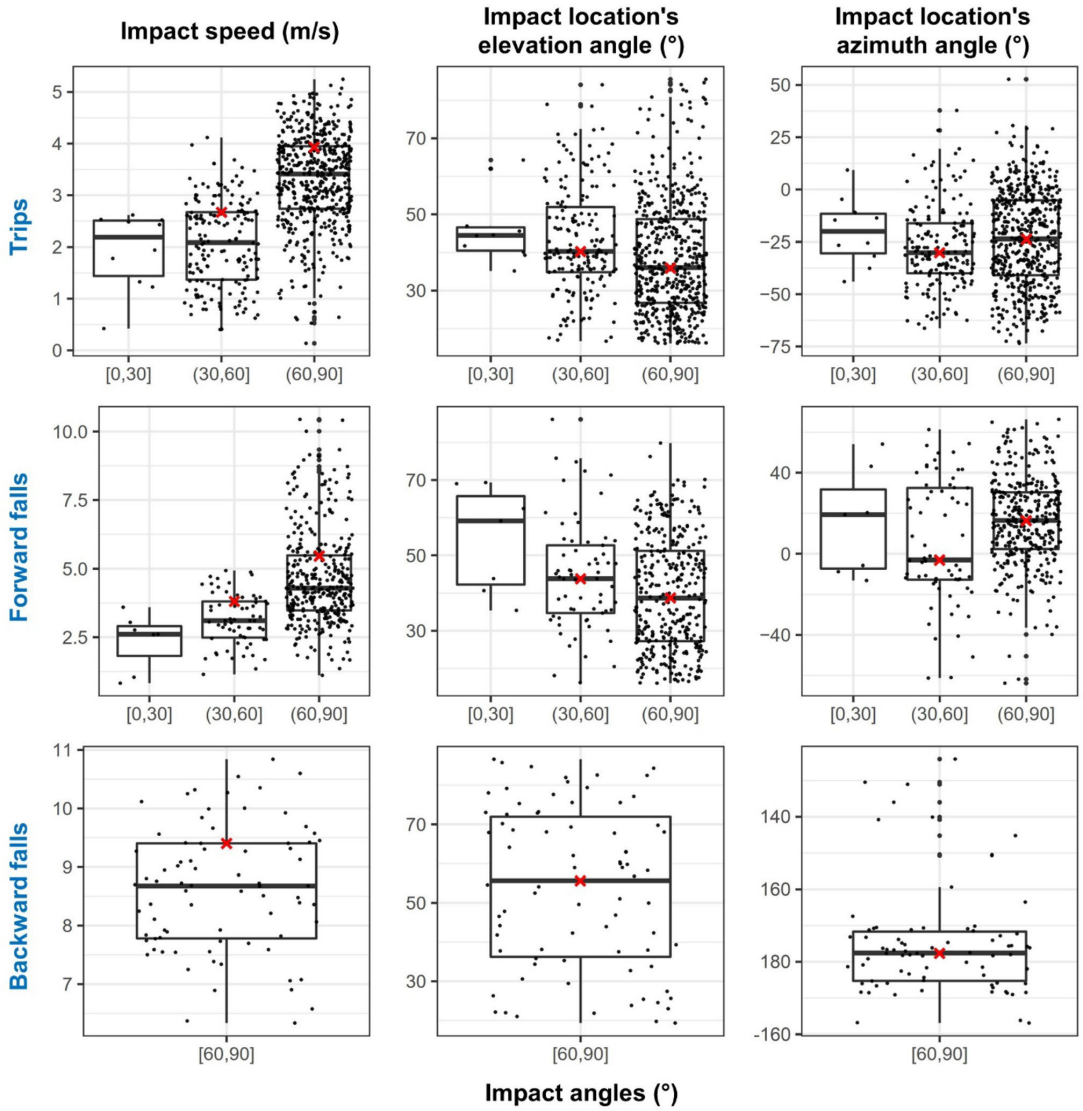
Distribution of the impact speed and elevation and azimuth angles with respect to three groups of impact angles (for cases with head impact within the helmet coverage area). The red crosses indicate the selected values for the representative head impact conditions.

As explained in the methods section, in each scenario, we excluded the cases that did not lead to a head impact or head impact occurred outside the helmet coverage area and plotted the distribution of the impact speed and elevation and azimuth angles with respect to three groups of impact angles, shown in Fig. 8. As can be seen in the figure, all backward falls led to impact angles between 60° and 90°. In addition, for the trips and forward falls, there are very few cases with an impact angle in the [0°, 30°] range (less than 2%). Hence, this shallow angle range was excluded from the following analysis, which determined head impact conditions for setting up representative lab tests. Another interesting observation in this figure is that the trips and forward falls with higher impact speeds have larger impact angles, thus being closer to normal impacts.

For each impact angle range, we determined the 75th percentile value of impact speed and 50th percentile value of elevation and azimuth angles. These conditions, summarised in Table 2, can be used to design tests that represent a wide range of impact conditions from trips and falls in workplace.

**TABLE 2:** The parameters of the five representative head impact conditions.

Impact conditions	Impact scenarios				
	Trip		Forward fall		Backward fall
Impact speed (m/s) [75th percentile value]	2.7	3.9	3.8	5.5	9.4
Impact angle (°) [mean value of angle range]	45	75	45	75	75
Elevation angle (°) [50th percentile value]	40.3	36.2	43.8	38.8	55.8
Azimuth angle (°) [50th percentile value]	− 30.2	− 23.7	− 2.9	16.5	177.5

## Discussion

We predicted the head impact location, speed and angle of a large range of trips and falls in the workplace. Trips and falls are the main causes of traumatic brain injuries in workers, but industrial helmets are not assessed for protection against impacts caused by them. We used multibody dynamics modelling to simulate trips and falls. This approach allowed us to study the effects of a range of parameters, including worker size, walking speed, bracing and falling height, on the head impact conditions. We determined a few head impact conditions representative of a large range of trips and falls, which can be used to design new industrial helmet test methods.

Our MADYMO simulations predicted large differences in the head impact speeds between trips and falls. The median head impact speed of trips was 2.9 m/s with arm bracing and 4.3 m/s without arm bracing. The impact speed of forward and backward falls increased significantly by increasing the falling height. For instance, the median head impact speeds were 3.5 and 8.1 m/s for forward falls from 0.5 and 4 m heights respectively. These median head impact speeds were higher in backward falls: 7.1 m/s (from 0.5 m height) and 9.5 m/s (from 4 m height). The higher impact speed in falls onto lower levels means much higher kinetic energy of the head prior to impact, which can lead to more severe head injuries compared with falls onto the same level (e.g., trips).^15^ This is in keeping with previous database analysis results, which showed that falls onto a lower level at workplace cause fatal head injuries while falls onto the same level are a common cause of non-fatal and mild head injuries.^8,9,11,36,45^

**FIGURE 9:**
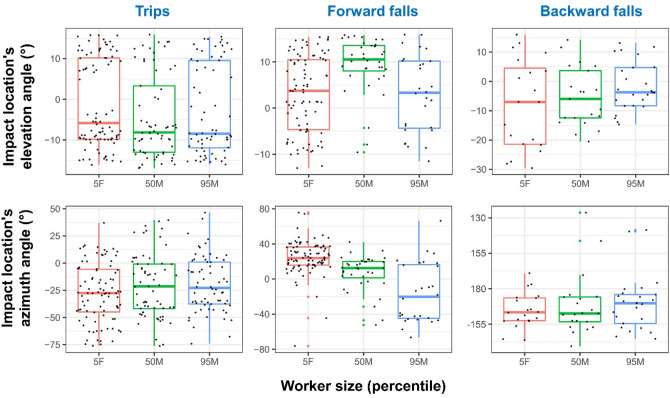
Distribution of the elevation and azimuth angles of impact locations with respect to the worker size (for cases with head impacts outside the helmet coverage area). For trips and backward falls, the median elevation angles of the three worker size group are negative, which suggests that the median impact locations are below the centre of gravity of human head. For forward falls, the median impact location is above the centre of gravity of human head. For trips and backward falls, the median distribution of azimuth angles are similar among different worker size groups. However, for forward falls, worker size has a significant effect on the distribution of azimuth angle.

The impact location predicted by simulations also agrees with findings in previous analyses of databases, which show that the frontal head region accounts for the largest portion (38–46%) of head impact locations in workplace accidents.^34^ Similarly, we have shown that the majority (55–68%) of the head impacts caused by trips and forward falls occur at the frontal region. Another interesting observation from our simulations is that 19–41% of impacts were outside the helmet coverage. Such head impacts may lead to wounds and superficial injuries. A recent database analysis showed that the majority (49.1% in Sweden and 61.4% in Germany) of head injuries are wounds and superficial injuries.^8^ This study did not include the location of these injuries, but our prediction of high percentage of impacts outside the helmet coverage may suggest that these injuries were mainly outside the helmet coverage area. And the distribution of these unprotected head impacts (See Appendix in Fig. 9) may indicate the location of such injury. This raises questions about the coverage area of helmets and calls for more studies and design interventions to avoid such injuries.

The simulations show that the mean head impact angle of different scenarios, i.e. trips, forward fall and backward fall, is 68°–84° from ground. These impact angles are much larger than those determined from motorcycle and bicycle collision reconstructions or simulations. For instance, an investigation of the head impact angle of 200 motorcycle collisions showed that 50% of the angles were less than 15° and 17.5% in the 16°–30° range.^10^ In addition, a reconstruction of 56 motorcycle collisions with other vehicles estimated a head impact angle of 44° ± 22°.^7^ Similarly, simulation of over 1000 cyclist falls showed that the head impact angle was between 32° and 56.5°.^6^ They showed that increasing the travelling speed from 5.5 to 11.1 m/s decreased the impact angle from 57° to 33°. Contrastingly, in our trip cases, increasing the walking speed resulted in an increase in the impact angle (Fig. 6) while in the forward and backward cases, the walking speed did not have a consistent effect on impact angle. This is likely due to the fact that workplace falls involve considerable body and ground interaction before the head-ground impact (Fig. 5b). For example, the tripping barrier constraints the foot producing rolling motion of the body, where the walking speed is transferred to the rolling speed. In such scenario, the head impact speed may have a higher normal component transferred from the rolling speed. A similar phenomenon has been observed in simulations of e-scooter falls, where increasing the travel speed led to a decrease in the head impact angle.^30^ In contrast, cyclist falls usually result in head-first impacts against the ground with limited body-ground interaction. Overall, we predicted larger head impact angles (68.5° to 84°) in workplace trips and falls than the previous work studying motorcycle collisions and cyclist falls.

For the first time, our study provides an estimation of the range of head impact speeds and angles in work-related fall accidents. Different countries have their own standard for industrial safety helmets, but their requirements for impact protection are very similar. These standards require preventions against heavy falling objects (shock absorption) and sharp falling objects (anti-penetration). For instance, the European standard for industrial safety helmets (EN397) requires a shock absorption test which involves impacting the helmet crown by a 5 kg rounded impactor at 4.4 m/s and an anti-penetration test which involves impacting the helmet crown by a 3 kg sharp impactor at 4.4 m/s. These tests represent impacts caused by heavy and sharp falling objects. However, being struck by/against an object only accounts for a small portion of accidents leading to work-related TBI while falls are the leading cause of TBI in workplace.^9,21,43^ To the best of our knowledge there is only one study that has evaluated industrial helmets in conditions similar to falls.^5^ However, they borrowed the impact speed and angle from studies on oblique impacts in sporting and road traffic collisions, thus not representing falls in the workplace.

The 75th percentile impact speeds predicted in trips and forward falls are 2.7 to 5.5 m/s, which are smaller than those of bicycle helmet tests, thus achievable with current helmet test rigs. However, this speed in the backward fall is 9.4 m/s, which exceeds the capacity of most helmet test rigs.^47^ Even if a test rig can reach this impact speed, conducting such high-speed impact tests on current industrial helmets is likely to damage the headform and sensors. Despite these difficulties, the head injury under such harsh impact conditions should not be ignored, particularly as data has shown that 80% of falls leading to TBI are from heights above 3 m.^36^ Future work is needed to address this issue through improving fall prevention measures in workplace and where falls are unavoidable, improving helmet design for protection against falls from height.

When determining the representative head impact conditions, we first plotted impact speed and azimuth and elevation angles against three groups of impact angles with a 30° intervals. This allowed us to exclude the groups that have very few cases and find a relationship between head impact speed and angle where most cases with higher impact speeds had higher impact angles. This is different to previous studies, which determined the mean or median values of each factor independently.^6,30^ The approach adopted here maintained the relationship between different factors and head impact angle with a 30° angle resolution, thus providing more comprehensive conditions for setting up helmet test methods representative of trips and falls in workplace.

This study has several limitations. First, the MADYMO human body models were primarily developed and validated to simulate occupant and pedestrian impacts in automotive industry. Here, we only validated the human body model against a backward fall workplace accident, not against the forward fall and trip accidents. This is because the backward fall accident was the only available and suitable CCTV footage we could find for validation. There are three reasons for the lack of suitable CCTV footage: (1) Although CCTV cameras are in place, they usually fail to capture workplace head injury accidents due to the obstruction of structures or equipment on site; (2) CCTV footages of workplace head injuries are usually inaccessible for privacy reasons; (3) Most captured workplace head injury accidents involve human–environment interactions. (e.g. human’s reaction of holding nearby structures to keep balance). Such CCTV footages cannot be used to validate the MADYMO passive human body models. Future work is required to further validate the MADYMO human body models when suitable video footage of forward falls and trips become available.

Secondly, human falls in the workplace have uncertainties, which can be affected by factors including impact media, human reaction, surrounding objects and more. A small change in these conditions can lead to very different head impact results.^1,13^ For instance, the difference in arm bracing gesture can lead to large differences in the outcome of head injury.^20^ It is not feasible to simulate all possible accident scenarios. In this work, we either simplified these conditions, such as arm bracing, or were not able to model them, such as human’s reaction of holding nearby structures to keep balance. This was due to the limited ability of the passive human models in MADYMO. Also, for this reason, we did not simulate fall scenarios with many interactions with surrounding objects. For instance, falls from ladders were excluded as available CCTV footages show that the head impact conditions are influenced by interaction between human body, ladder steps and other objects, such as an unstable ground support.^3^ Further studies using other methods, such as analysis of CCTV footage, are required to estimate the head impact conditions in more complicated fall scenarios.

Another limitation is the determination of the backward walking speeds, which were measured from a small group of volunteers without considering the human size group (e.g. 5th female and 50th male), workplace environment (e.g. floor condition and obstruction) and loading conditions (e.g. walking backwards while holding heavy objects). The measured backward walking speeds may not be representative of those of industrial workers in real-world working environment. Therefore, we selected six backward walking speeds (between 0.2 and 1.2 m/s), aiming to represent a wider range of backward walking scenarios and mitigate the potential bias in the underrepresentation of human size.

In summary, we studied the head impact conditions by simulating typical trip and fall cases, covering a large number of possible accident situations relevant to the workplace. We found that:

1. A large proportion of trips and falls result in head impacts outside the normal region of helmet coverage.
2. Trips and forward falls lead to head impacts mostly in front region while most backward falls produce head impacts to the back and top region.
3. Almost all initial conditions have significant effects on head impact speed and angle, but this depends on different scenarios. For example, the worker size does not have a significant effect on the head impact speed and angle in trips, but it significantly affects the head impact speed and angle in forward and backward falls.
4. The workplace falls lead to head impacts with larger impact angles than observed in motorcycle and bicycle collisions, with the majority of trips and forward falls producing head impacts with an angle larger than 30° and backward falls producing head impacts with an angle larger than 60°.
5. The impact speed ranges from 2.7 to 9.4 m/s, with trips producing lowest and backward falls producing highest speeds.
6. The five representative head impact conditions (Table 2) can represent a large proportion of head impacts caused by the simulated falls and trips.

The head impact conditions determined here can be used to evaluate the performance of industrial helmets, provide a guideline for future industrial helmet test methods and inform improvements in standards with the aim of mitigating head and brain injuries in workplace falls.

## Appendix

See Table 3 and Fig. 9.

**Table 3:** Effect of initial conditions on head impact speed and angle.

Initial conditions	Trips		Forward falls to lower level		Backward falls to lower level	
	Head impact speed	Head impact angle	Head impact speed	Head impact angle	Head impact speed	Head impact angle
Worker size	F(2, 1139) = 1.14, *p* = 0.32	F(2, 1139) = 1.975, *p* = 0.139	F(2, 567) = 55.5, *p* < 0.001	F(2, 567) = 16.24, *p* < 0.001	F(2, 141) = 6.85, *p* < 0.01	F(2, 141) = 0.77, *p* = 0.464
Walking stances	F(2, 1139) = 41.77, *p* < 0.001	F(2, 1139) = 124.7, *p* < 0.001	–	–	–	–
Walking velocity	F(5, 1136) = 21.02, *p* < 0.001	F(5, 1136) = 3.65, *p* < 0.01	F(5, 564) = 7.08, *p* < 0.001	F(5, 564) = 10.13, *p* < 0.001	F(5, 138) = 11.42, *p* < 0.001	F(5, 138) = 15.16, *p* < 0.001
Tripping barrier width	F(2, 1139) = 5.877, *p* < 0.01	F(2, 1139) = 5.517, *p* < 0.01	–	–	–	–
Tripping barrier height	F(3, 1138) = 15.54, *p* < 0.001	F(3, 1138) = 7.37, *p* < 0.001	–	–	–	–
Arm bracing	F(1, 1140) = 326.9, *p* < 0.001	F(1, 1140) = 155.4, *p* < 0.001	–	–	–	–
Falling height	–	–	F(7, 562) = 60.29, *p* < 0.001	F(7, 562) = 9.68, *p* < 0.001	F(7, 136) = 12.03, *p* < 0.001	F(7, 136) = 3.90, *p* < 0.001
Foot to edge distance	–	–	F(3, 566) = 21.84, *p* < 0.001	F(3, 566) = 1.51, *p* = 0.21	–	–
